# Supplementary material for: A numerical approach applied to three-dimensional wave scattering problems subjected to obliquely propagating incident waves
Source: PLoS One. 2024 Jun 6;19(6):e0304721. doi: 10.1371/journal.pone.0304721 (PMC11156327; doi:10.1371/journal.pone.0304721)
Supplement: Attachment — Submitted filename: Review PONE-D-24-11204.pdf [file pone.0304721.s003.pdf]

Manuscript ID: PONE-D-24-11204

Title: *Implementation and Application of a Method for Three-dimensional Wave Scattering Problem with Arbitrary Incident Angle of Waves*

**Summary:** Accurate modeling of artificial boundary conditions and wave inputs is vital for numerical simulations of wave scattering in semi-infinite domains. Traditional methods focus on one or two-dimensional free-field problems, limiting their ability to handle arbitrary incident directions in three-dimensional scenarios. This paper proposes a novel approach for inputting plane seismic waves from any direction. It employs a combination of transfer matrix analysis for two-dimensional free-field problems and transformation between coordinate systems for three-dimensional motion. This method, integrated with the Finite Element Method (FEM), facilitates input for various boundaries. Numerical examples demonstrate its effectiveness in analyzing seismic responses, particularly in nuclear power plants and deep-water bridges, confirming its accuracy in handling complex scenarios.

**Observations** & **Suggestions**: For the analysis of three-dimensional fluctuation scattering problems, the conventional approach typically employs free-field analysis in one or two dimensions, often focusing on perpendicular or parallel incidence relative to one of the coordinate planes containing the vertical axis. However, this method restricts the incident directions of fluctuations in three-dimensional space and inadequately captures the true three-dimensional effects of ground vibration input. To address this limitation, it becomes imperative to analyze the free field at arbitrary fluctuation incident directions in three-dimensional space, providing inputs for the comprehensive analysis of fluctuation scattering in three-dimensional problems.

Huang (2015) proposed a solution for homogeneous half-space-free field incidents in any direction by combining the direct incident wave with the reflected wave from the ground surface. Nevertheless, when tackling the free field analysis of horizontally layered sites, this method encounters challenges in tracking the complex reflected and transmitted waves, thereby intensifying the difficulty of solving the problem. To make the kinds of literature sections richer the authors are advised to include the following references:

Barak, M. S., Kumar, M., Kumari, M., & Singh, A. (2020). Inhomogeneous wave propagation in partially saturated soils. *Wave Motion*, *93*, 102470.

Barak, M. S., Kumari, M., & Kumar, M. (2018). Effect of local fluid flow on the propagation of plane waves at an interface of water/double-porosity solid with an underlying uniform elastic solid. *Ocean Engineering*, *147*, 195-205.

Kumari, M., Barak, M. S., & Kumar, M. (2017). Seismic reflection and transmission coefficients of a single layer sandwiched between two dissimilar poroelastic solids. *Petroleum Science*, *14*(4), 676-693.

**Recommendations:** The work done in this manuscript is appreciable, I recommend the manuscript for possible publication with incorporating the suggestions.
