# Supplementary material for: A numerical approach applied to three-dimensional wave scattering problems subjected to obliquely propagating incident waves
Source: PLoS One. 2024 Jun 6;19(6):e0304721. doi: 10.1371/journal.pone.0304721 (PMC11156327; doi:10.1371/journal.pone.0304721)
Supplement: Attachment — Submitted filename: Comments to the authors - PONE-D-24-11204.doc [file pone.0304721.s002.doc]

In this paper, a novel method was proposed for inputting plane seismic wave incidents. Initially, the two-dimensional free-field problem was analyzed using the transfer matrix method. Then, a three-dimensional free-field motion was calculated by transforming between two coordinate systems. However, there are several issues that must be addressed by the authors before the paper is to be published. These comments are listed as follows:

1- It is recommended to express the sentences of the paper in the third person. Presenting these items is not particularly appropriate in the abstract section.
2- The abstract could be written better. The innovation of the study as well as the step-by-step research process should be mentioned in it.
3- As can be seen (Section 2.1, lines 143 to 146), the approach used in the paper is a numerical method. If so, it is recommended to mention this issue in the title of the manuscript. For example, the title can be modified as follows: “A numerical approach applied to three-dimensional wave scattering problems subjected to obliquely propagating incident waves”.
4- In section 2, line 130, it is mentioned that an analytical response is used! It is mandatory to mention the reference.
5- The author has well pointed out the capability and efficiency of the used approach. In order to complete the paper, it is recommended to mention some limitations of the method used at the end of the conclusion section.
6- The number of paragraphs in the paper is very high. It is better to reduce its number and edit it.
7- The section of the conclusion is written very poorly. The authors should revise this section.
8- Some references in wave propagation problems by other numerical methods (such as the boundary element method) are missed. Here are some papers the authors should have known:

<https://link.springer.com/article/10.1007/s11803-020-0603-9>

<https://link.springer.com/article/10.1007/s41062-019-0257-5>

<https://www.equsci.org.cn/en/article/doi/10.29382/eqs-2018-0137-3>

<https://www.tandfonline.com/doi/abs/10.1080/13632469.2021.1927886>

9- The paper requires extensive editorial work.
