# Supplementary material for: A numerical approach applied to three-dimensional wave scattering problems subjected to obliquely propagating incident waves
Source: PLoS One. 2024 Jun 6;19(6):e0304721. doi: 10.1371/journal.pone.0304721 (PMC11156327; doi:10.1371/journal.pone.0304721)
Supplement: Attachment — Submitted filename: Response to Reviewers.doc [file pone.0304721.s004.doc]

**Response to Reviewers**

Thank you very much for giving us the opportunity to revise the manuscript. We have carefully read the comments and tried our best to modify the manuscript. The revised parts are marked in red. Our response to the comments of the reviewers is as follows.

# Review Comments to the Author

Please use the space provided to explain your answers to the questions above. You may also include additional comments for the author, including concerns about dual publication, research ethics, or publication ethics. (Please upload your review as an attachment if it exceeds 20,000 characters)

**Reviewer #1**: In this paper, a novel method was proposed for inputting plane seismic wave incidents. Initially, the two-dimensional free-field problem was analyzed using the transfer matrix method. Then, a three-dimensional free-field motion was calculated by transforming between two coordinate systems. However, there are several issues that must be addressed by the authors before the paper is to be published. These comments are listed as follows:

# Response to Reviewer #1:

Thank you for your time and patience. We made changes practically exactly as you suggested. In the revised manuscript, we highlight in red font the added text in response to the reviewers’ comments. We hope we addressed all your concerns.

**1***- It is recommended to express the sentences of the paper in the third person. Presenting these items is not particularly appropriate in the abstract section.*

**Response:** Thank you for your time and patience. We followed your suggestion and modified and polished the language of the full text. The revised parts are marked in red.

**2***- The abstract could be written better. The innovation of the study as well as the step-by-step research process should be mentioned in it.*

**Response:** Thank you. As per your guidance, we have reformulated the abstract section, elucidating the novelty and the incremental research process therein. The revised parts are marked in red.

On line 22 page 2, we rewrote this paragraph as follows:

“Accurately modeling artificial boundary conditions and wave inputs is paramount for numerical simulations of wave scattering in semi-infinite domains within seismic engineering. Traditionally, analysts focused on one- or two-dimensional free-field problems to determine wave inputs, primarily for vertically incident plane waves or obliquely incident waves parallel to two axes. However, these methods were inadequate for handling arbitrary incident directions in three-dimensional scenarios. This paper proposes a method for modeling seismic wave incidents in arbitrary directions. The basic theory of viscoelastic boundaries is leveraged, and a plane containing an arbitrary incident direction and the vertical coordinate axis is selected to establish a two-dimensional plane coordinate system. The two-dimensional free-field problem in this coordinate system is derived using the transfer matrix method. Subsequently, displacement, velocity, and stress are converted into the coordinate system where the three-dimensional calculation model is located, providing input for the three-dimensional scattering problem. Furthermore, the implementation of transmitting boundary conditions and viscoelastic boundary wave inputs is presented to enable incident wave scattering problems at any angle of the plane. The effect of oblique-incidence soil-structure dynamic interaction is also discussed, focusing on the parallel technology method adopted in this paper. With the relatively mature technology route and method, together with nuclear power systems and large-span deep-water bridge models, through examples of comparative analysis, qualitative and quantitative analyses are made on the impact on the soil mass, foundation, and structure when the seismic wave is an oblique incident.”

**3***- As can be seen (Section 2.1, lines 143 to 146), the approach used in the paper is a numerical method. If so, it is recommended to mention this issue in the title of the manuscript. For example, the title can be modified as follows: “A numerical approach applied to three-dimensional wave scattering problems subjected to obliquely propagating incident waves”.*

**Response:** Thank you.

Following your advice, we believe that the title you suggested better encapsulates the core issue of this paper: the implementation of numerical methods. The original title may have leaned more towards the seismic wave incidence problem using arbitrary angle methods, which might not be sufficiently comprehensive. Therefore, we have revised the paper title according to your recommendation.

**4***- In section 2, line 130, it is mentioned that an analytical response is used! It is mandatory to mention the reference.*

**Response:** Thanks. As per your suggestion, we have added relevant references here and labelled them in the text.

On line 126 page 6, we add the following reference:

[30] Thomson W T. Transmission of Elastic Waves through a Stratified Solid Medium. Journal of Applied Physics. 1950; 21(2):89-93. https://doi.org/10.1063/1.1699629

[31] Haskell, N. The dispersion of surface waves on multilayer media. Bull.Seism.Soc.AM 1953; 43: 17-34. https://doi.org/10.1785/BSSA0430010017

[33] Liao Z P. Introduction to wave motion theories in engineer. Science Press, 2002. (in chinese).

**5***- The author has well pointed out the capability and efficiency of the used approach. In order to complete the paper, it is recommended to mention some limitations of the method used at the end of the conclusion section.*

**Response:** Thanks for your comments. Thank you. We followed your suggestion and modified the end section. The revised parts are marked in red.

On line 686 page 33, we rewrote this paragraph as follows:

“While this paper proposed a computational method under the seismic wave input from any direction and validates its feasibility and effectiveness through numerical examples. Considering the complexity of seismic wave propagation in the site, such as non-uniform seismic input, the influence of the seismic source, and site nonlinearity, the accuracy of the computed results still requires comparison and verification with engineering experiments and field monitoring data. Therefore, subsequent Verification and Validation (V&V) are necessary to ensure the accuracy of the computational method.”

**6***- The number of paragraphs in the paper is very high. It is better to reduce its number and edit it.*

**Response:** Thank you for your time and patience. Following your advice, we have merged relevant paragraphs in the paper, reducing the overall number of paragraphs.

**7**- *The section of the conclusion is written very poorly. The authors should revise this section.*

**Response:** Thank you very much for bringing our attention to the structures of “Conclusion”. We followed your suggestion and modified and polished the language of the conclusion. The revised parts are marked in red.

On line 657 page 32, we rewrote this paragraph as follows:

“This paper presents a numerical wave analysis of the scattering of plane waves incident from any spatial direction by combining the transmission boundary and viscoelastic boundary simulations of wave scattering problems. Utilizing the transfer matrix method and coordinate transformation, the free field when a plane wave was incident in any direction was obtained. This approach incorporates the concentrated mass explicit finite element method to achieve numerical wave analysis of plane wave scattering problems with incidence from any spatial direction. Through numerical examples, the effectiveness of this method was validated, highlighting the significant influence of the incident angle on seismic response. Through numerical computations, the following conclusions have been derived:

(1) This paper utilizes the MPI protocol and FORTRAN language to implement a parallel scheme for the dynamic analysis of three-dimensional time-domain SSI under spatial seismic wave incidence.
(2) Under the influence of obliquely incident seismic waves, structures exhibit varying patterns in acceleration response corresponding to changes in the angle of incidence. Under SV wave incidents, when the incident angle *θ*_2_ = 0°, a comparative analysis reveals no time difference in the displacement response. However, when the second spatial incidence angle *θ*_2_ ≠ 0°, the vibration onset in all three directions and the arrival time of peak displacement response were delayed, consistent with theoretical calculations.
(3) As the seismic wave incidence angle increases, the response of the near-source side abutment increases while that of the far-source side abutment decreases. The displacement response of the main girder in the approach bridge decreases with the oblique incidence angle, particularly when the incidence angle was perpendicular to the valley slope, resulting in the same peak displacement response for the main girder in the approach bridge near the source. The observed responses of the bridge under seismic wave action conform to the expected patterns, thus validating the accuracy of the proposed methodology from a practical perspective.

While this paper proposed a computational method under the seismic wave input from any direction and validates its feasibility and effectiveness through numerical examples. Considering the complexity of seismic wave propagation in the site, such as non-uniform seismic input, the influence of the seismic source, and site nonlinearity, the accuracy of the computed results still requires comparison and verification with engineering experiments and field monitoring data. Therefore, subsequent Verification and Validation (V&V) are necessary to ensure the accuracy of the computational method.”

**8**- *Some references in wave propagation problems by other numerical methods (such as the boundary element method) are missed. Here are some papers the authors should have known:*

<https://link.springer.com/article/10.1007/s11803-020-0603-9>

<https://link.springer.com/article/10.1007/s41062-019-0257-5>

<https://www.equsci.org.cn/en/article/doi/10.29382/eqs-2018-0137-3>

<https://www.tandfonline.com/doi/abs/10.1080/13632469.2021.1927886>

<https://link.springer.com/article/10.1007/s11803-020-0603-9>

<https://link.springer.com/article/10.1007/s41062-019-0257-5>

<https://www.equsci.org.cn/en/article/doi/10.29382/eqs-2018-0137-3>

<https://www.tandfonline.com/doi/abs/10.1080/13632469.2021.1927886>

**Response:** Thank you. I apologize for the inconvenience, but I need to further strengthen my understanding of the outcomes related to using numerical methods to solve wave propagation problems. Following your suggestion, I have incorporated citations to the relevant literature at the appropriate places in the text (References [13] and [17]). Thank you for your recommendation.

[13] Panji, M., Ansari, B. Anti-plane seismic ground motion above twin horseshoe-shaped lined tunnels. Innov. Infrastruct. Solut. 2020; 5: 7. https://doi.org/10.1007/s41062-019-0257-5

[17] Panji, M., Habibivand, M. Seismic analysis of semi-sine shaped alluvial hills above subsurface circular cavity. Earthq. Eng. Eng. Vib. 2020; 19: 903-917. https://doi.org/10.1007/s11803-020-0603 -9

**9**- *The paper requires extensive editorial work.*

**Response:** Thank you for your time and patience. We followed your suggestion and modified and polished the language of the full text. The revised parts are marked in red.

**Reviewer #2**:

**Summary***: Accurate modeling of artificial boundary conditions and wave inputs is vital for numerical simulations of wave scattering in semi-infinite domains. Traditional methods focus on one or two-dimensional free-field problems, limiting their ability to handle arbitrary incident directions in three-dimensional scenarios. This paper proposes a novel approach for inputting plane seismic waves from any direction. It employs a combination of transfer matrix analysis for two-dimensional free-field problems and transformation between coordinate systems for three-dimensional motion. This method, integrated with the Finite Element Method (FEM), facilitates input for various boundaries. Numerical examples demonstrate its effectiveness in analyzing seismic responses, particularly in nuclear power plants and deep-water bridges, confirming its accuracy in handling complex scenarios.*

**Observations & Suggestions***: For the analysis of three-dimensional fluctuation scattering problems, the conventional approach typically employs free-field analysis in one or two dimensions, often focusing on perpendicular or parallel incidence relative to one of the coordinate planes containing the vertical axis. However, this method restricts the incident directions of fluctuations in three-dimensional space and inadequately captures the true three-dimensional effects of ground vibration input. To address this limitation, it becomes imperative to analyze the free field at arbitrary fluctuation incident directions in three-dimensional space, providing inputs for the comprehensive analysis of fluctuation scattering in three-dimensional problems.*

*Huang (2015) proposed a solution for homogeneous half-space-free field incidents in any direction by combining the direct incident wave with the reflected wave from the ground surface. Nevertheless, when tackling the free field analysis of horizontally layered sites, this method encounters challenges in tracking the complex reflected and transmitted waves, thereby intensifying the difficulty of solving the problem. To make the kinds of literature sections richer the authors are advised to include the following references:*

*Barak, M. S., Kumar, M., Kumari, M., & Singh, A. (2020). Inhomogeneous wave propagation in partially saturated soils. Wave Motion, 93, 102470.*

*Barak, M. S., Kumari, M., & Kumar, M. (2018). Effect of local fluid flow on the propagation of plane waves at an interface of water/double-porosity solid with an underlying uniform elastic solid. Ocean Engineering, 147, 195-205.*

*Kumari, M., Barak, M. S., & Kumar, M. (2017). Seismic reflection and transmission coefficients of a single layer sandwiched between two dissimilar poroelastic solids. Petroleum Science, 14(4), 676-693.*

**Recommendations***: The work done in this manuscript is appreciable, I recommend the manuscript for possible publication with incorporating the suggestions.*

# Response to Reviewer #2:

**Response:** Thank you for carefully reading our manuscript and for your suggestions on how to improve it. I apologize for the inconvenience, but I need to further strengthen my understanding of the outcomes related to using numerical methods to solve wave propagation problems. Following your suggestion, I have incorporated citations to the relevant literature at the appropriate places in the text (References [27] and [28]). Thank you for your recommendation.

[27] Kumari, M., Barak, M. S., Kumar, M. Seismic reflection, and transmission coefficients of a single layer sandwiched between two dissimilar poroelastic solids. Petroleum Science. 2017; 14(4): 676-693. https://doi.org/10.1007/s12182-017-0195-9

[28] Barak, M. S., Kumar, M., Kumari, M., Singh, A. Inhomogeneous wave propagation in partially saturated soils. Wave Motion.2020;93:102470. <https://doi.org/10.1016/j.wavemoti.2019>. 102470
