# Supplementary material for: A numerical approach applied to three-dimensional wave scattering problems subjected to obliquely propagating incident waves
Source: PLoS One. 2024 Jun 6;19(6):e0304721. doi: 10.1371/journal.pone.0304721 (PMC11156327; doi:10.1371/journal.pone.0304721)
Supplement: Attachment — Submitted filename: Response to Reviewers.doc [file pone.0304721.s005.doc]

**Response to Reviewers**

Thank you very much for giving us the opportunity to revise the manuscript. We have carefully read the comments and modified the manuscript. The revised parts are marked in red. Our response to the comments of the reviewers is as follows:

**Response to Reviewer:**

*1.* ***Please include a figure label and title for Figures to 1 to 33 in your main manuscript.***

**On line 694, page 33,** **we have added figure labels and titles as follows:**

**S1 Fig. Schematic diagram of soil structure interaction model under spatial incidence of seismic wave.**

**S2 Fig. Geometrical relation of the multi-transmitting boundary.**

**S3 Fig. Schematic diagram of the finite calculation area and equivalent spring-damper system on the artificial boundary.**

**S4 Fig. Equal force and stress direction of interfaces in all directions.**

**S5 Fig. Finite element model of the site.**

**S6 Fig. Input wave.**

**S7 Fig. Displacement of half space subjected to SV wave oblique incidence.**

**S8 Fig. The horizontal displacement time history of reference point A under SV wave oblique incidence.**

**S9 Fig. An example model schematic diagram.**

**S10 Fig. Input plus wave.**

**S11 Fig. Displacement in *y*-direction at observation points. Note: T and V represent the transmitting boundary and the viscous-spring artificial boundary.**

**S12 Fig. Displacement of the observation points (SH wave).**

**S13 Fig. Displacement of the observation points.**

**S14 Fig. An example model schematic diagram of the NPP.**

**S15 Fig. The input wave and the cut-off frequency.**

**S16 Fig. Schematic diagram of the partitioned analysis of soil-structure interaction and the position of the reference points of the soil subdomain.**

**S17 Fig. Displacement response of soil reference points A_11_ and A_31_.**

**S18 Fig. Displacement response of soil reference points A_13_ and A_33_.**

**S19 Fig. Displacement response of soil reference points A_21_ and A_23_.**

**S20 Fig. Displacement response of the foundation reference point A_22_.**

**S21 Fig. The response of the structure.**

**S22 Fig. General layout of the bridge.**

**S23 Fig. Finite element model of the bridge.**

**S24 Fig. Acceleration response spectrum.**

**S25 Fig. Ground motion and its Fourier amplitude spectrum.**

**S26 Fig. Schematic diagram of the calculation site.**

**S27 Fig. Displacement at the bottom of pier 9 (*x*-direction).**

**S28 Fig. Peak displacement response at the bottom of the abutment (*x*-direction).**

**S29 Fig. Displacement response of approach bridge (*x*-direction).**

**S30 Fig. Peak displacement response at the bearing of the main girder of the approach bridge (*x*-direction).**

**S31 Fig. Displacement response of approach bridge (*x*-direction).**

**S32 Fig. Time history of shear force at the bottom of the abutment (*x*-direction).**

**S33 Fig. Peak shear force at the bottom of the abutment (*x*-direction).**

**S1 Data. (Zip) Original data for figures in the article.**

Additionally, we have renamed the images and data in the supplementary files **Figures.zip** and **S1 Data.zip** to comply with the journal's requirements.

We appreciate your review and valuable suggestions once again. Should you have any further recommendations or requests regarding our modifications, please feel free to inform us.
