# Supplementary material for: A numerical approach applied to three-dimensional wave scattering problems subjected to obliquely propagating incident waves
Source: PLoS One. 2024 Jun 6;19(6):e0304721. doi: 10.1371/journal.pone.0304721 (PMC11156327; doi:10.1371/journal.pone.0304721)
Supplement: Attachment — Submitted filename: Response to Reviewers.doc [file pone.0304721.s006.doc]

**Dear Dr. S. M. Anas,**

Academic Editor

PLOS ONE

I apologize for taking up so much of your time. Thank you very much for carefully reviewing our manuscript, providing positive feedback, and offering suggestions for revision.

I would like to clarify that regarding the issues raised in the second revised manuscript titled "A numerical approach applied to three-dimensional wave scattering problems subjected to obliquely propagating incident waves" [PONE-D-24-11204R1], we have made modifications and provided explanations in our response to the first round of minor revisions. The revised manuscript was completed on May 11th and returned as requested. Subsequently, on May 13th, we received the request to add figure labels and captions for Figures 1 to 33. We have made the necessary changes and returned the manuscript accordingly. In this revision, we replaced the response to the May 11th modifications (regarding the addition or removal of references) with the response addressing the modification of "figure labels and captions," which may have caused you to overlook our relevant statements and feedback on your suggestions. We sincerely apologize for this oversight. Therefore, in this response, we will combine the feedback from the last two rounds of revisions and provide a unified reply.

Thank you again for your evaluation of our revised manuscript and for providing valuable feedback.

Best regards,

**Dr. Hao Lv**

College of Safety and Environmental Engineering, Shandong University of Science and Technology, Qingdao 266590, China

The relevant modification responses are as follows：

**Response to Reviewers**

Thank you for your evaluation of our revised manuscript and for providing valuable feedback. In response to your guidance, we have carefully reviewed the suggested references and reassessed their relevance to our research.

During the review process, we indeed noted the issue you mentioned regarding the addition of suggested references without providing relevant justifications, which was an inappropriate practice. Consequently, we have conducted a comprehensive review of the literature and confirmed its alignment with the focus of our study.

We acknowledge the importance of ensuring the relevance of references to the research topic in enhancing the quality of the manuscript. Hence, we have removed references that are not pertinent to our study. The revised parts are marked in red.

**Response to Reviewer（first** **minor revised manuscript，May 11th）:**

*Additional Editor Comments:*

*Dear Authors,*

*I hope this email finds you well.*

*I am writing to inform you about the status of your revised manuscript entitled "A numerical approach applied to three-dimensional wave scattering problems subjected to obliquely propagating incident waves" [PONE-D-24-11204R1]. The revised version was sent to the previous reviewers for reevaluation, and I'm pleased to inform you that both reviewers recommended the paper for publication and expressed satisfaction with your responses to their comments.*

*However, upon my preliminary assessment of the revised manuscript, I observed that you have added references suggested by the reviewers without providing a relevant reason for their inclusion. Therefore, I have decided to request a minor revision. I kindly ask you to review the suggested references and determine whether they are truly relevant to your study. If they are not relevant, please remove them. If they are relevant, kindly provide a valid reason for their addition.*

*Please address this revision request at your earliest convenience and resubmit the manuscript accordingly. Feel free to reach out if you have any questions or need further clarification.*

*Thank you for your attention to this matter.*

*Best regards,*

*Dr. S. M. Anas*

*Academic Editor*

*PLOS ONE*

**And the third minor revised manuscript，May 15th**

*Additional Editor Comments:*

*Dear Authors,*

*I hope this email finds you well.*

*I am writing to inform you about the status of your second revised manuscript titled "A numerical approach applied to three-dimensional wavescattering problems subjected to obliquely propagating incident waves" [PONE-D-24-11204R2]. Upon review of the second revision, it has come toour attention that the minor revision requested in the previous communication has not been adequately addressed in your author response form.*

*In our previous correspondence, I had requested a minor revision concerning the inclusion of references suggested by the reviewers withoutproviding a relevant reason for their addition. Although your second revised manuscript has been submitted, the concerns outlined in the previousrevision request remain unaddressed.*

*Therefore, I kindly ask you to carefully review the comments provided by the editor and ensure that all requested revisions are addressedappropriately in your resubmission. Specifically, please provide valid reasons for the inclusion of any additional references suggested by thereviewers.*

*We understand that revisions can be time-consuming, but it is essential to ensure the quality and relevance of the manuscript before proceedingwith the publication process. Your prompt attention to this matter would be greatly appreciated.*

*Should you have any questions or require further clarification regarding the revision process, please do not hesitate to contact us.*

*Thank you for your cooperation and understanding.*

*Best regards,*

*Dr. S. M. Anas*

*Academic Editor*

*PLOS ONE*

**Our response to the comments of the reviewers is as follows:**

The number of lines and pages indicated here are those in reference file **PONE-D-24-11204_R1.pdf**

**1- On line 56, page 3,**

Based on your suggestion, we have re-evaluated the relevant content of the paper, and have decided to remove reference [13]. The primary rationale behind this decision is the document's emphasis on engineering practice, which is not closely related to the theoretical research on site response analysis in this section. After careful consideration, we have opted to exclude reference [13].

**Reference:**

[13] Panji, M., Ansari, B. Anti-plane seismic ground motion above twin horseshoe-shaped lined tunnels. Innov. Infrastruct. Solut. 2020; 5: 7. https://doi.org/10.1007/s41062-019-0257-5

**2- On line 62, page 3,**

In response to the reviewer's suggestions, reference [17] has been incorporated. This decision was driven by the referenced study's utilization of the half-plane time domain boundary element method (BEM) to conduct seismic analysis on semi-sine shaped alluvial hills above circular underground cavities. The study elucidated the significant impact of the hill's material properties and its heterogeneity with the underlying half-space on surface response. This falls within the realm of site analysis methodologies, aligning with the thematic content of this section and enriching the scope of theoretical exploration. The authors deemed its inclusion appropriate.

**Reference:**

[17] Panji, M., Habibivand, M. Seismic analysis of semi-sine shaped alluvial hills above subsurface circular cavity. Earthq. Eng. Eng. Vib. 2020; 19: 903-917. https://doi.org/10.1007/s11803-020-0603 -9

**3- On line 85, page 4,**

References [27,28] primarily explore the seismic reflection and transmission characteristics between two dissimilar poroelastic solids, as well as the propagation of inhomogeneous plane harmonic waves in dissipatively partially saturated soils. The research focuses on solving and analyzing the response of saturated soil sites. While this provides valuable insights for extending our research focus to saturated soil sites, it has limited relevance to the theoretical analysis of site response in this section. Therefore, we have decided to cautiously remove references [27,28].

**Reference:**

[27] Kumari, M., Barak, M. S., Kumar, M. Seismic reflection, and transmission coefficients of a single layer sandwiched between two dissimilar poroelastic solids. Petroleum Science. 2017; 14(4): 676-693. https://doi.org/10.1007/s12182-017-0195-9

[28] Barak, M. S., Kumar, M., Kumari, M., Singh, A. Inhomogeneous wave propagation in partially saturated soils. Wave Motion.2020;93:102470. <https://doi.org/10.1016/j.wavemoti.2019. 102470>

**4- On line 126, page 6,** we add the following reference:

[30] Thomson W T. Transmission of Elastic Waves through a Stratified Solid Medium. Journal of Applied Physics. 1950; 21(2):89-93. https://doi.org/10.1063/1.1699629

[31] Haskell, N. The dispersion of surface waves on multilayer media. Bull.Seism.Soc.AM 1953; 43: 17-34. https://doi.org/10.1785/BSSA0430010017

[33] Liao Z. P. Introduction to wave motion theories in engineer. Science Press, 2002. (in chinese).

Following the recommendations of the reviewers and after careful consideration, references [30, 31], and [33] are included in this section. Professor Thomson and Professor Haskell are acknowledged pioneers in the field of engineering theory. The referenced studies in these citations have addressed the propagation issues of waves in site conditions and have provided analytical solutions, thus possessing significant insights and reference value. Therefore, references [30] and [31] have been incorporated at this juncture.

Academician Liao's reference [33] focuses predominantly on discussing practical artificial boundary conditions for simulating infinite domains, thereby systematically expounding on numerical simulation techniques for near-field wave propagation. This technique finds application not only in addressing solid dynamics problems in civil engineering, mechanical engineering, and geophysics but also holds utility for tackling linear and nonlinear dynamics issues in large-scale complex systems and the near-field challenges associated with elastic wave scattering. Moreover, it offers reference value for investigating near-field wave propagation problems in technology domains such as electromagnetics and acoustics. Additionally, the author's research group maintains a fruitful collaboration with Academician Liao, who has provided valuable insights and suggestions for the author's relevant research endeavors.

**Following the revision of the relevant literature content, the corresponding references have been renumbered and appropriately modified.**

**Response to Reviewer（second minor revised manuscript，May 13th）:**

*1.* ***Please include a figure label and title for Figures to 1 to 33 in your main manuscript.***

In the “Supporting Information” section of the article, we have added figure labels and titles as follows:

**S1 Fig. Schematic diagram of soil structure interaction model under spatial incidence of seismic wave.**

**S2 Fig. Geometrical relation of the multi-transmitting boundary.**

**S3 Fig. Schematic diagram of the finite calculation area and equivalent spring-damper system on the artificial boundary.**

**S4 Fig. Equal force and stress direction of interfaces in all directions.**

**S5 Fig. Finite element model of the site.**

**S6 Fig. Input wave.**

**S7 Fig. Displacement of half space subjected to SV wave oblique incidence.**

**S8 Fig. The horizontal displacement time history of reference point A under SV wave oblique incidence.**

**S9 Fig. An example model schematic diagram.**

**S10 Fig. Input plus wave.**

**S11 Fig. Displacement in *y*-direction at observation points. Note: T and V represent the transmitting boundary and the viscous-spring artificial boundary.**

**S12 Fig. Displacement of the observation points (SH wave).**

**S13 Fig. Displacement of the observation points.**

**S14 Fig. An example model schematic diagram of the NPP.**

**S15 Fig. The input wave and the cut-off frequency.**

**S16 Fig. Schematic diagram of the partitioned analysis of soil-structure interaction and the position of the reference points of the soil subdomain.**

**S17 Fig. Displacement response of soil reference points A_11_ and A_31_.**

**S18 Fig. Displacement response of soil reference points A_13_ and A_33_.**

**S19 Fig. Displacement response of soil reference points A_21_ and A_23_.**

**S20 Fig. Displacement response of the foundation reference point A_22_.**

**S21 Fig. The response of the structure.**

**S22 Fig. General layout of the bridge.**

**S23 Fig. Finite element model of the bridge.**

**S24 Fig. Acceleration response spectrum.**

**S25 Fig. Ground motion and its Fourier amplitude spectrum.**

**S26 Fig. Schematic diagram of the calculation site.**

**S27 Fig. Displacement at the bottom of pier 9 (*x*-direction).**

**S28 Fig. Peak displacement response at the bottom of the abutment (*x*-direction).**

**S29 Fig. Displacement response of approach bridge (*x*-direction).**

**S30 Fig. Peak displacement response at the bearing of the main girder of the approach bridge (*x*-direction).**

**S31 Fig. Displacement response of approach bridge (*x*-direction).**

**S32 Fig. Time history of shear force at the bottom of the abutment (*x*-direction).**

**S33 Fig. Peak shear force at the bottom of the abutment (*x*-direction).**

**S1 Data. (Zip) Original data for figures in the article.**

Additionally, we have renamed the images and data in the supplementary files **Figures.zip** and **S1 Data.zip** to comply with the journal's requirements.

**We appreciate your review and valuable suggestions once again. Should you have any further recommendations or requests regarding our modifications, please feel free to inform us.**
